# Risk Factors for Hospitalization Due to Community-Acquired Sepsis – A Population-Based Case-Control Study

**DOI:** 10.1371/journal.pone.0124838

**Published:** 2015-04-21

**Authors:** Daniel Pilsgaard Henriksen, Anton Pottegård, Christian B. Laursen, Thøger Gorm Jensen, Jesper Hallas, Court Pedersen, Annmarie Touborg Lassen

**Affiliations:** 1 Department of Emergency Medicine, Odense University Hospital, DK-5000 Odense C, Denmark; 2 Clinical Pharmacology, Department of Public Health, University of Southern Denmark, DK-5000 Odense C, Denmark; 3 Department of Respiratory Medicine, Odense University Hospital, DK-5000 Odense C, Denmark; 4 Department of Clinical Microbiology, Odense University Hospital, DK-5000 Odense C, Denmark; 5 Department of Clinical Chemistry & Pharmacology, Odense University Hospital, DK-5000 Odense C, Denmark; 6 Department of Infectious Diseases, Odense University Hospital, DK-5000 Odense C, Denmark; D'or Institute of Research and Education, BRAZIL

## Abstract

**Background:**

The aim of the study was to estimate risk factors for hospitalization due to sepsis and to determine whether these risk factors vary by age and gender.

**Methods:**

We performed a population-based case-control study of all adult patients admitted to a medical ED from September 2010 to August 2011. Controls were sampled within the hospital catchment-area. All potential cases were manually validated using a structured protocol. Vital signs and laboratory values measured at arrival were registered to define systemic inflammatory response syndrome and organ dysfunction. Multivariable logistic regression was used to elucidate which predefined risk factors were associated with an increased or decreased risk hospitalization due to sepsis.

**Results:**

A total of 1713 patients were admitted with sepsis of any severity. The median age was 72 years (interquartile range: 57–81 years) and 793 (46.3%) were male. 621 (36.3%) patients were admitted with sepsis, 1071 (62.5%) with severe sepsis and 21 (1.2%) with septic shock. Episodes with sepsis of any severity were associated with older age (85+ years adjusted OR 6.02 [95%CI: 5.09–7.12]), immunosuppression (4.41 [3.83–5.09]), alcoholism-related conditions (2.90 [2.41–3.50]), and certain comorbidities: psychotic disorder (1.90 [1.58–2.27]), neurological (1.98 [1.73–2.26]), respiratory (3.58 [3.16–4.06]), cardiovascular (1.62 [1.41–1.85]), diabetes (1.82 [1.57–2.12]), cancer (1.44 [1.22–1.68]), gastrointestinal (1.71 [1.44–2.05]) and renal (1.46 [1.13–1.89]). The strength of the observed associations for comorbid factors was strongest among younger individuals.

**Conclusions:**

Hospitalization due to sepsis of any severity was associated with several independent risk factors, including age and comorbid factors.

## Introduction

Severe sepsis is a life-threatening condition defined by a systemic inflammatory response (SIRS) caused by infection, hypoperfusion and organ dysfunction[1]. The syndrome is a common cause of admission at the emergency department (ED)[2]. While the majority of sepsis studies mainly have focused on the acute management of the disease in order to reduce the mortality[3,4], recent studies have changed this focus to a more preventive health view on sepsis[5–7] identifying patients at risk of admission with sepsis. Several risk factors have been identified including chronic medical conditions[5,7–9], old age[7,10], male gender[9,11,12], alcoholism-related conditions[8,13], immunosuppression[14], and possibly also use of antipsychotic medication[15]. We aimed to examine the risk factors for hospitalization with community-acquired sepsis of any severity based on a manually validated case material, and to determine whether these risk factors vary by age and gender.

## Materials and Methods

We conducted a population-based case control study of all patients hospitalized to a medical ED with sepsis from 1 September 2010–31 August 2011. We used admissions to a large primary hospital with tertiary functions to identify cases and national health registries to identify population-based controls. As virtually all medical care in Denmark is furnished by the national health authorities, these data resources allow true population-based studies covering all inhabitants in the selected region of Denmark[16].

### Ethics Statement

In compliance with Danish law, the study was notified to and approved by the Danish Data Protection Agency (J No 2008-58-0035), and the access to patient clinical records was approved by the Danish National Board of Health (J No 3-3013-35). No further ethical approval, or consent from participants, is needed for register-based studies in Denmark. Data were anonymised and de-identified prior data analysis.

### Study design and setting

The study was conducted as a population-based case-control study where both cases and controls were sampled from Odense University Hospitals’ catchment-area. Cases, defined as patients hospitalized with sepsis of any severity, were compared to controls, to elucidate which factors were associated with being a case, i.e. were associated with an increased or decreased risk of hospitalization due to sepsis.

Odense University Hospital is a tertiary academic hospital with all specialities represented. Although the hospital provides high level care for persons living in the region of South Denmark (pop: 1.2 million) it also serves as the primary care hospital for all residents in the hospital catchment-area of 235,000 adults.

The medical ED served as a medical admission unit for the following medical specialities: general internal medicine, infectious diseases; gastroenterology; geriatric medicine; rheumatology; endocrinology and respiratory medicine. The medical ED received all acutely hospitalized medical patients from either primary care or the open general ED. The open general ED serves as a level-1 trauma centre with mixed medical-surgical patients.

### Cases

Baseline characteristics of the case material are described in detail elsewhere[17]. Briefly, eligible patients were all adults (≥15 years) admitted to the medical ED or directly to the medical ICU. Patients, who died in the hospital’s open general ED before transfer to the medical ED, were also considered eligible. Patients were included as cases the first time they presented with sepsis of any severity within the study period.

Time of arrival to the hospital, clinical information, and vital signs at the time of admission were electronically extracted from the patient’s records and validated by trained data abstractors with the aim to identify cases.

In order to identify patients with community-acquired infection, we manually reviewed all admissions to the medical ED or directly to the medical ICU within the study period (N = 8358). We used a structured protocol, based on The National Healthcare Safety Network criteria[18] in combination with a predefined definition, requiring that the site of infection was clinically evident. Two experienced clinicians (DPH and CBL) assessed the inter-observer reliability by analyzing 209 randomly selected patients from all admissions to the medical ED (N = 8358), and found a general inter-rater agreement, regarding the presence of infection, of 84.1% (κ 0.68), corresponding to a substantial strength of agreement[19].

In order to exclude possible hospital-acquired infections we excluded cases with a prior hospitalization up to 7 days before the current admission. Patients transferred from other hospitals, patients residing outside the hospitals catchment-area at the time of admission and patients who were unidentified throughout the entire course of admission were excluded as well.

### Controls

As controls, we sampled all adults (≥15 years) with residence in the hospital catchment-area (N = 235,598) in the study period. Each subject was assigned a random index date during the study period, and if the subject was a resident that day, had not been hospitalized up to 7 days before the index date, and had not previously been included as case, then the subject was included as control.

### Risk factors/ Covariates

We examined the following potential risk factors for hospitalization with sepsis: age; gender; a history of alcoholism-related conditions; immunosuppression and chronic medical conditions including a history of psychotic disorder as described in the following.

### Data sources

By using the unique Danish personal identification number assigned to all Danish residents[16], we were able to retrieve information from several population-based registers to identify the predefined risk factors. The Danish Civil Registration System was used to retrieve data on births, deaths, and migration status[20]. The Danish National Patient Register was used to retrieve data on previous discharge diagnoses[21]. Odense Pharmacoepidemiological Database was used to retrieve data on redeemed prescriptions[22]. The Danish National Cancer Register was used to retrieve data on cancer diagnoses[23], and the Danish National Alcohol- and Drug Treatment Register to retrieve data on alcohol treatment.

### Definitions

Patients were categorized as having sepsis, severe sepsis or septic shock at arrival to the hospital according to the ACCP/SCCM published criteria[24]. Severe sepsis was defined as sepsis and at least one organ dysfunction not previously observed/recognized (S1 Appendix).

Comorbidity is reported based on discharge diagnoses the last 10 years up to 7 days prior to the current admission or index date for cases and controls. To avoid the possibility of a strong correlation in the covariates cancer and immunosuppression, we defined cancer in the “cancer comorbidity” category as a stable state (from 10–1 years prior the index date), whereas active cancer (new cancer diagnosis from 365–7 days prior the index date) was included in the immunosuppression covariate. We defined alcohol-induced liver disease (ICD-10: K70*) as alcoholism-related conditions and excluded these ICD-10 codes from the “Gastrointestinal” comorbidity definition. We defined a history of psychotic disorder by one or more redeemed prescriptions of antipsychotic medication (ATC: N05A*) from 2007 and up to the current admission for cases, or index date for controls (S1 Appendix).

### Analysis

Age was categorized in three categories (15–64, 65–84 and 85+ years). We tested if age could be included as a continuous variable in the model using fractional polynomials, but age violated the assumption of linearity[25]. Also, due to the low number of patients admitted with septic shock (N = 21) we combined the severe sepsis and septic shock categories.

In the main analysis, the outcome of interest was sepsis of any severity and exposure of interest was the predefined potential risk factors. For all risk factors, three analyses were performed: 1) A crude, univariate analysis looking at the single risk factor, 2) A multivariate analysis for the single risk factor adjusted for gender and age, 3) A multivariable analysis adjusted for all of the remaining potential risk factors (fully adjusted model).

In order to identify potential effect measure modification we repeated the analyses stratified by gender and age.

Similarly, to assess whether the effect of the single risk factors varied when examining patients with sepsis or severe sepsis, we repeated the fully adjusted model in these two groups separately.

Data were presented as medians with interquartile range (IQR) or proportions with 95% confidence intervals (95%CI) calculated under the assumption of a binomial distribution. Missing data was treated as within normal range if data were continuous and defined as within the reference category if data were categorized.

All calculations were performed using Stata Release 13.0 (StataCorp, College Station, TX, USA).

## Results

### Patient characteristics

A total of 8358 admissions to the medical ED or directly to the ICU within the study period, 3605 admissions (43.1%) where the patient had signs of an infection and 2490 (29.8%) met the criteria for sepsis of any severity. In total we excluded 730 due to: re-admissions with sepsis (N = 377), living outside catchment-area (N = 209) and discharged <7 days prior inclusion (N = 144). We ended up with 1713 patients with a first-time admission with sepsis of any severity within the study period.

The median age of the included patients was 72 years (IQR: 57–81 years) and 793 (46.3%) were male. 621 (36.3%) presented with sepsis, 1071 (62.5%) presented with severe sepsis and 21 (1.2%) presented with septic shock (Table 1).

**Table 1:** Characteristics of patients hospitalized to a medical emergency department with sepsis of any severity, sepsis and severe sepsis.

		Sepsis of any severity	Sepsis	Severe sepsis	Septic shock
	Total	(n = 1,713)	(n = 621)	(n = 1,071)	(n = 21)
Gender	Female	920 (53.7%)	348 (56.0%)	564 (52.7%)	8 (38.1%)
	Male	793 (46.3%)	273 (44.0%)	507 (47.3%)	13 (61.9%)
Admission at entry	Directly to the medical ED	1,098 (64.1%)	377 (60.7%)	709 (66.2%)	12 (57.1%)
	Open general ED	590 (34.4%)	242 (39.0%)	340 (31.7%)	8 (38.1%)
	Directly to ICU	25 (1.5%)	2 (0.3%)	22 (2.1%)	1 (4.8%)
Sites of infection per patient, N	1	1,453 (84.8%)	548 (88.2%)	891 (83.2%)	14 (66.7%)
	2	242 (14.1%)	67 (10.8%)	169 (15.8%)	6 (28.6%)
	3	18 (1.1%)	6 (1.0%)	11 (1.0%)	1 (4.8%)
Sites of infection	Central nervous system	18 (1.1%)	9 (1.4%)	9 (0.8%)	0 (0.0%)
	Lower respiratory tract	1,077 (62.9%)	335 (53.9%)	734 (68.5%)	8 (38.1%)
	Urinary tract	415 (24.2%)	143 (23.0%)	266 (24.8%)	6 (28.6%)
	Abdominal	184 (10.7%)	77 (12.4%)	102 (9.5%)	5 (23.8%)
	Cardiovascular	11 (0.6%)	6 (1.0%)	5 (0.5%)	0 (0.0%)
	Skin, muscles, bones	98 (5.7%)	52 (8.4%)	42 (3.9%)	4 (19.0%)
	Viral/systemic	42 (2.5%)	17 (2.7%)	24 (2.2%)	1 (4.8%)
	Unknown with bacteremia	23 (1.3%)	4 (0.6%)	17 (1.6%)	2 (9.5%)
	Unknown without bacteremia	75 (4.4%)	34 (5.5%)	39 (3.6%)	2 (9.5%)
	Other	39 (2.3%)	21 (3.4%)	18 (1.7%)	0 (0.0%)
Bacteremia	No	1,541 (90.0%)	590 (95.0%)	938 (87.6%)	13 (61.9%)
	Yes	172 (10.0%)	31 (5.0%)	133 (12.4%)	8 (38.1%)
Number of organ failures	0	621 (36.3%)	621 (100.0%)	0 (0.0%)	0 (0.0%)
	1	651 (38.0%)	0 (0.0%)	651 (60.8%)	0 (0.0%)
	2	300 (17.5%)	0 (0.0%)	299 (27.9%)	1 (4.8%)
	3+	141 (8.2%)	0 (0.0%)	121 (11.3%)	20 (95.2%)
Site of organ failure	CNS	333 (19.4%)	0 (0.0%)	321 (30.0%)	12 (57.1%)
	Metabolic	226 (13.2%)	0 (0.0%)	209 (19.5%)	17 (81.0%)
	Cardiovascular	100 (5.8%)	0 (0.0%)	79 (7.4%)	21 (100.0%)
	Respiratory	709 (41.4%)	0 (0.0%)	697 (65.1%)	12 (57.1%)
	Renal	106 (6.2%)	0 (0.0%)	99 (9.2%)	7 (33.3%)
	Hepatic	55 (3.2%)	0 (0.0%)	48 (4.5%)	7 (33.3%)
	Coagulation	209 (12.2%)	0 (0.0%)	195 (18.2%)	14 (66.7%)

^a^The number could exceed the total number of cases, because a patient could have more than one site of infection or site of organ failure.

### Risk factors of hospitalization associated with sepsis of any severity

All of the potential risk factors were associated with a significantly increased risk of hospitalization with sepsis of any severity in the univariate risk factor estimates (Table 2). In the adjusted regression models, all risk factors except gender showed statistically significant associations (Table 2). Old age 85+ years (Odds ratio (OR) 6.02, 95%CI: 5.09–7.12) and immunosuppression (OR 4.41, 95%CI: 3.83–5.09) exhibited the strongest associations with incident sepsis of any severity in the fully adjusted model (Table 2).

**Table 2:** Demographic characteristics and risk of hospitalization with community-acquired sepsis of any severity.

	Controls	Cases	Crude OR^a^ [95%CI]	Adjusted OR [95%CI]^b^	Adjusted OR [95%CI]^c^
Gender					
Female	115,773	920	1.00 (ref.)	1.00 (ref.)	1.00 (ref.)
Male	111,281	793	0.90 [0.81–0.99]	1.07 [0.97–1.18]	1.01 [0.91–1.11]
Age groups (yrs)					
15–64	178,902	633	1.00 (ref.)	1.00 (ref.)	1.00 (ref.)
65–84	42,296	815	5.53 [4.98–6.14]	5.54 [4.99–6.15]	3.09 [2.75–3.48]
85+	5,856	265	13.35 [11.54–15.44]	13.47 [11.63–15.61]	6.02 [5.09–7.12]
Immunosuppression					
No	223,137	1,359	1.00 (ref.)	1.00 (ref.)	1.00 (ref.)
Yes	3,917	354	16.21 [14.36–18.31]	9.44 [8.31–10.73]	4.41 [3.83–5.09]
Alcoholism-related conditions					
No	221,464	1,555	1.00 (ref.)	1.00 (ref.)	1.00 (ref.)
Yes	5,590	158	4.11 [3.48–4.86]	5.11 [4.31–6.06]	2.90 [2.41–3.50]
*Comorbidities*					
Psychotic disorder					
No	220,977	1,550	1.00 (ref.)	1.00 (ref.)	1.00 (ref.)
Yes	6,077	163	3.90 [3.31–4.59]	3.03 [2.57–3.59]	1.90 [1.58–2.27]
Neurological					
No	217,469	1,324	1.00 (ref.)	1.00 (ref.)	1.00 (ref.)
Yes	9,585	389	6.91 [6.16–7.75]	2.95 [2.60–3.35]	1.98 [1.73–2.26]
Respiratory					
No	217,201	1,206	1.00 (ref.)	1.00 (ref.)	1.00 (ref.)
Yes	9,853	507	9.72 [8.74–10.80]	6.99 [6.27–7.79]	3.58 [3.16–4.06]
Cardiovascular					
No	218,210	1,337	1.00 (ref.)	1.00 (ref.)	1.00 (ref.)
Yes	8,844	376	7.20 [6.41–8.09]	3.25 [2.87–3.69]	1.62 [1.41–1.85]
Diabetes					
No	219,055	1,458	1.00 (ref.)	1.00 (ref.)	1.00 (ref.)
Yes	7,999	255	4.91 [4.29–5.62]	3.07 [2.67–3.52]	1.82 [1.57–2.12]
Cancer					
No	218,665	1,510	1.00 (ref.)	1.00 (ref.)	1.00 (ref.)
Yes	8,389	203	3.57 [3.08–4.14]	1.76 [1.51–2.05]	1.44 [1.22–1.68]
Gastrointestinal					
No	222,544	1,533	1.00 (ref.)	1.00 (ref.)	1.00 (ref.)
Yes	4,510	180	5.99 [5.12–7.01]	3.52 [2.99–4.15]	1.71 [1.44–2.05]
Renal					
No	225,259	1,631	1.00 (ref.)	1.00 (ref.)	1.00 (ref.)
Yes	1,795	82	6.56 [5.23–8.23]	3.24 [2.56–4.10]	1.46 [1.13–1.89]

^a^OR = odds ratio

^b^Adjusted for gender and age

^c^Adjusted for a history of alcoholism-related conditions, comorbidity divided into specific organ systems, gender, age in age categories (15–64, 65–84 and 85+ years) and immunosuppression.

### Sepsis severity

Differences in the strength of association between patients hospitalized with sepsis and severe sepsis include age and psychotic disorder, where patients hospitalized with severe sepsis had a higher odds of being older and with a history of psychotic disorder. The lower 95% confidence intervals of the adjusted OR for cancer and renal comorbidity, for patients with sepsis were just below 1 (cancer: OR 1.30 [95%CI 0.99–1.72]; renal: OR 1.46 [95%CI 0.95–2.23]) compared to severe sepsis (cancer: OR 1.47 [95%CI 1.22–1.78]; renal: OR 1.37 [95%CI 1.01–1.85]). See Table 3 for more details.

**Table 3:** Demographic characteristics and risk of hospitalization with community-acquired sepsis and severe sepsis.

		Sepsis			Severe sepsis		
		Cases	Crude OR [95%CI]	Adjusted OR [95%CI]^a^	Cases	Crude OR [95%CI]	Adjusted OR [95%CI]^a^
Sex	Female	348	1.00 (ref.)	1.00 (ref.)	572	1.00 (ref.)	1.00 (ref.)
	Male	273	0.82 [0.70–0.96]	0.89 [0.76–1.05]	520	0.95 [0.84–1.07]	1.07 [0.95–1.22]
Age groups (years)	15–64	291	1.00 (ref.)	1.00 (ref.)	342	1.00 (ref.)	1.00 (ref.)
	65–84	254	3.71 [3.13–4.39]	2.15 [1.78–2.60]	561	7.02 [6.13–8.03]	3.93 [3.39–4.56]
	85+	76	8.07 [6.26–10.40]	3.66 [2.74–4.88]	189	17.41 [14.55–20.84]	7.84 [6.38–9.63]
Immunosuppression	No	498	1.00 (ref.)	1.00 (ref.)	871	1.00 (ref.)	1.00 (ref.)
	Yes	123	16.11 [13.18–19.68]	5.03 [3.98–6.34]	221	17.01 [14.61–19.79]	4.45 [3.73–5.30]
Alcoholism-related conditions	No	568	1.00 (ref.)	1.00 (ref.)	983	1.00 (ref.)	1.00 (ref.)
	Yes	53	3.68 [2.77–4.88]	2.64 [1.94–3.59]	109	4.41 [3.61–5.38]	2.93 [2.34–3.67]
*Comorbidities*							
Psychotic disorder	No	579	1.00 (ref.)	1.00 (ref.)	971	1.00 (ref.)	1.00 (ref.)
	Yes	42	2.69 [1.96–3.68]	1.35 [0.97–1.88]	121	4.67 [3.86–5.65]	2.26 [1.83–2.78]
Neurological	No	502	1.00 (ref.)	1.00 (ref.)	822	1.00 (ref.)	1.00 (ref.)
	Yes	119	5.43 [4.44–6.64]	1.90 [1.52–2.38]	270	7.64 [6.65–8.78]	1.93 [1.65–2.25]
Respiratory	No	442	1.00 (ref.)	1.00 (ref.)	764	1.00 (ref.)	1.00 (ref.)
	Yes	179	9.07 [7.62–10.81]	3.70 [3.01–4.54]	328	9.76 [8.56–11.12]	3.29 [2.82–3.84]
Cardiovascular	No	509	1.00 (ref.)	1.00 (ref.)	828	1.00 (ref.)	1.00 (ref.)
	Yes	112	5.49 [4.47–6.74]	1.46 [1.15–1.86]	264	8.08 [7.02–9.29]	1.65 [1.40–1.94]
Diabetes	No	554	1.00 (ref.)	1.00 (ref.)	904	1.00 (ref.)	1.00 (ref.)
	Yes	67	3.33 [2.58–4.30]	1.33 [1.01–1.75]	188	5.81 [4.96–6.81]	2.02 [1.70–2.41]
Cancer	No	560	1.00 (ref.)	1.00 (ref.)	950	1.00 (ref.)	1.00 (ref.)
	Yes	61	2.85 [2.19–3.72]	1.30 [0.99–1.72]	142	3.95 [3.30–4.71]	1.47 [1.22–1.78]
Gastrointestinal	No	570	1.00 (ref.)	1.00 (ref.)	963	1.00 (ref.)	1.00 (ref.)
	Yes	51	4.45 [3.34–5.94]	1.39 [1.02–1.91]	129	6.78 [5.62–8.16]	1.82 [1.48–2.24]
Renal	No	595	1.00 (ref.)	1.00 (ref.)	1,036	1.00 (ref.)	1.00 (ref.)
	Yes	26	5.55 [3.74–8.24]	1.46 [0.95–2.23]	56	6.97 [5.30–9.16]	1.37 [1.01–1.85]

^a^Adjusted for a history of alcoholism-related conditions, comorbidity divided into specific organ systems, gender, age in age categories (15–64, 65–84 and 85+ years) and immunosuppression.

### Gender- and age stratified analysis

We found no significant difference in risk factors when stratifying by gender, except the distribution of age in the age categories, where the risk of hospitalization of older males was significantly higher compared to females in the same age category (85+ years) (Table 4).

**Table 4:** Adjusted odds ratios^a^ for admission with sepsis of any severity stratified by gender.

	Females		Males	
	N, females	Adjusted OR	N, males	Adjusted OR
Age groups (yrs)				
15–64	346/88,565	1.00 [Ref]	287/89,704	1.00 [Ref]
65–84	419/22,346	2.31 [1.97–2.71]	396/19,135	3.02 [2.54–3.58]
85+	155/3,942	3.68 [2.97–4.56]	110/1,649	6.86 [5.32–8.84]
Immunosuppression				
No	706/112,676	1.00 [Ref]	653/109,102	1.00 [Ref]
Yes	214/2,177	5.50 [4.63–6.54]	140/1,386	4.83 [3.92–5.94]
Alcoholism-related conditions				
No	851/112,949	1.00 [Ref]	704/106,960	1.00 [Ref]
Yes	69/1,904	3.21 [2.46–4.20]	89/3,528	2.63 [2.07–3.33]
Number of concurrent comorbidities				
0	292/93,216	1.00 [Ref]	244/90,386	1.00 [Ref]
1–2	301/16,624	3.69 [3.11–4.37]	262/15,259	3.72 [3.09–4.49]
>2	327/5,013	8.04 [6.66–9.70]	287/4,843	7.56 [6.16–9.28]

^a^Adjusted for a history of alcoholism-related conditions, the sum of comorbidities: (Psychotic disorder, Neurological, Respiratory, Cardiovascular, Diabetes, Cancer, Gastrointestinal, Renal) age in age categories (15–64, 65–84 and 85+ years) and immunosuppression

Females in the young age category were more likely to be hospitalized with sepsis of any severity compared to males in the same age category, whereas males were more likely to hospitalized in the 85+ age category. The association between immunosuppression as well as the sum of comorbidities with hospitalization with sepsis of any severity were less pronounced with increased age (Table 5).

**Table 5:** Adjusted odds ratios^a^ for admission with sepsis of any severity stratified by age in age categories.

	15–64 years		65–84 years		85+ years	
	Cases / Controls	Adjusted OR^a^	Cases / Controls	Adjusted OR^a^	Cases / Controls	Adjusted OR^a^
Gender						
Female	346/88,565	1.00 [Ref]	419/22,346	1.00 [Ref]	155/3,942	1.00 [Ref]
Male	287/89,704	0.83 [0.71–0.97]	396/19,135	1.05 [0.91–1.21]	110/1,649	1.55 [1.20–2.01]
Immunosuppression						
No	528/176,663	1.00 [Ref]	609/39,856	1.00 [Ref]	222/5,259	1.00 [Ref]
Yes	105/1,606	9.54 [7.54–12.07]	206/1,625	4.71 [3.96–5.61]	43/332	2.45 [1.72–3.50]
Alcoholism-related conditions						
No	534/173,720	1.00 [Ref]	763/40,638	1.00 [Ref]	258/5,551	1.00 [Ref]
Yes	99/4,549	3.45 [2.72–4.37]	52/843	1.91 [1.41–2.58]	7/40	2.54 [1.10–5.90]
Number of concurrent comorbidities						
0	318/156,119	1.00 [Ref]	162/25,124	1.00 [Ref]	56/2,359	1.00 [Ref]
1–2	195/18,924	3.96 [3.29–4.76]	277/11,116	3.41 [2.80–4.16]	91/1,843	2.01 [1.43–2.82]
>2	120/3,226	9.32 [7.33–11.86]	376/5,241	8.12 [6.69–9.86]	118/1,389	3.07 [2.20–4.27]

^a^Adjusted for a history of alcoholism-related conditions, the sum of comorbidities: (Psychotic disorder, Neurological, Respiratory, Cardiovascular, Diabetes, Cancer, Gastrointestinal, Renal) gender and immunosuppression.

## Discussion

In this large population-based cohort study we found several independent risk factors associated to hospitalization at a medical ED with community-acquired sepsis of any severity. We found a large difference in the contribution of the individual risk factors between young and old patients.

We found that the risk factor most strongly associated to hospitalization with sepsis of any severity was old age. Increasing age has previously been associated with hospitalization with severe sepsis, both in studies focusing on age stratified incidence rates[11,26–29] as well as in studies depicting risk factors in multivariate models[5,7,27].

The comorbid condition exhibiting the strongest association with an incident hospitalization with sepsis of any severity was chronic respiratory disease with an adjusted OR 3.56. This is in accordance with a study by Wang et al (OR 1.5)[5]. The difference in OR estimates between the studies is likely due to different classifications or case identification method. Wang et al used self-reported pulmonary medication as a proxy for chronic respiratory disease, whereas we used discharge diagnoses based on a validated score system[30,31].

To account for potential clinically meaningful predefined effect modifiers, we stratified by age and gender and applied to a multivariate model. The association of the different risk factors was different when comparing the young and old patients. Like a previous study we found that female gender was a risk factor in the youngest age categories but was protective among the oldest[32]. This could be due to the different sites of infection dominating in the youngest age categories causing admission with sepsis, where especially urinary tract infections were associated with female gender in the young age groups (data not shown).

The strength of immunosuppression and most of the evaluated comorbidities as risk factors for hospitalization with sepsis of any severity showed a distinct variation by age with the highest OR in youngest age category and lowest among the oldest. This could either be due to survivor bias (healthy survivor effect), where patients who had lived to old age have lived with their comorbid condition for so long, that the effect as a risk factor for admission with sepsis decreases. Another possible explanation is, that age is such a strong risk factor for hospitalization with sepsis, that other risk factors are less important.

An interesting finding was, that the risk factors’ strength of association were in large parts similar among patients hospitalized due to sepsis and severe sepsis. Only old age, and psychotic disorder were stronger risk factors of hospitalization due to severe sepsis compared to sepsis. This similarity between the severity of sepsis could be due to the structure of the Danish healthcare system in regard to acute care. All patients included in the study were seen by either an ED physician or a general practitioner, who decided that the patient was in need of an admission to the medical ED. If we included all contacts from the open general ED (including those who were sent home without being admitted), perhaps this would have lead to more differentiated risk factors strength of association in the different severity groups, but this is beyond the scope of the study.

Information regarding the risk factors and mortality is important for several reasons. It is important to identify the characteristics of individuals at increased risk of developing a given illness, when devising disease risk stratification or prevention strategies. Public health benefits have, over the last century, resulted from evidence-based risk stratification for conditions like cardiovascular disease, and it is a hope that it is possible for sepsis as well[33,34].

For the physician working at an ED, knowledge of risk factors could potentially aid in earlier identification of patients with sepsis compared to patients hospitalized to the medical ED due to conditions other than sepsis, but this would require another selection of controls than we chose, as it was not the aim of the study.

Other risk factors have previously been evaluated, including marital status, where hospitalization with sepsis are more common among single, widowed, and legally separated individuals[35]. Further socioeconomic status (low education level and low income) have been associated with a future risk of sepsis[5].

### Strengths

Due to the uniformly organized Danish public healthcare system we could identify all patients included in the study in the different population-based registers for a full medical record. We used manual chart review with a structured protocol to collect data regarding the presence of infections at the time of admission, and based our study on symptoms and clinical findings at the time of arrival to the hospital.

### Limitations

The current work was a single-centre study from a medical ED at a university hospital, which potentially could affect the generalizability of the study. However, the hospital is the only hospital in the area and is a primary hospital for all residents in the hospital catchment-area, in parallel to highly specialized tertiary functions. Therefore we do not expect that residents of the catchment-area to be admitted to other hospitals.

The study was conducted at a medical ED describing acutely hospitalized medical patients. As such, the risk factors’ strength of association might be different among patients hospitalized to an abdominal surgery department, or patients in active chemotherapy. This could also lead to an underreporting of the number of patients hospitalized due to sepsis, and underestimate the risk factors strength of association among the chronic medical conditions associated with admission to departments not included in the current study.

Subsequent validation of the cohort identified by the review, yielded a kappa value of 0.67. This shows, that even when a well-defined classification of infection is applied, it is difficult to obtain a high inter-rater agreement.

Another limitation is that hospitalisation is a complex outcome with multiple components. There is the emergence of the medical condition itself, sepsis, but also extraneous factors such as the patient’s decision to seek medical attention, the patient’s follow-though in acquiring it and the physician’s decision to hospitalise. Depending on the circumstances, some of the extraneous factors may play an important role in the outcome and thus make the interpretation of our results challenging.

The seven day exclusion period is shorter compared to other definitions of hospital acquired infections. However, we did not find any significant differences in the risk factors strength of association when using a 30 day exclusion period (data not shown).

There is always the possibility of residual confounding, which could lead to different estimates when interpreting the independent risk factors in the multivariate models. Potential confounders could include socioeconomic status, and atrial fibrillation[5,36].

## Conclusions

We found that age, comorbidity including a history of psychotic disorder, immunosuppression and a history of alcoholism-related conditions served as independent risk factors for hospitalization with sepsis of any severity. We found a large difference in the risk factors’ strength of association in the different age categories, where comorbid conditions displayed stronger associations among young patients.

## Supporting Information

S1 Appendix Definitions of comorbidity, alcohol-related conditions, immunosuppression, infection, bacteremia, and organ dysfunction. (DOCX)
