# Supplementary material for: Risk Factors for Hospitalization Due to Community-Acquired Sepsis – A Population-Based Case-Control Study
Source: PLoS One. 2015 Apr 21;10(4):e0124838. doi: 10.1371/journal.pone.0124838 (PMC4405362; doi:10.1371/journal.pone.0124838)
Supplement: S1 Appendix — (DOCX) [file pone.0124838.s001.docx]

## Definitions

### Comorbidity

Discharge diagnoses from the Danish National Patient Register from 10 years prior admission/index date to 7 days prior.

**Cardiovascular disease:**

Myocardial infarction ICD-10: I21;I22;I23

Congestive heart failure ICD-10: I50; I11.0; I13.0; I13.2

Peripheral vascular disease ICD-10: I70; I71; I72; I73; I74; I77

**Neurological disease:**

Cerebrovascular disease ICD-10: I60-I69; G45; G46

Dementia ICD-10: F00-F03; F05.1; G30

Hemiplegia ICD-10: G81; G82

**Respiratory disease**

Chronic pulmonary disease ICD-10: J40-J47; J60-J67; J68.4; J70.1; J70.3; J84.1; J92.0; J96.1; J98.2; J98.3

**Diabetes mellitus with- or without chronic complications**

ICD-10: E10.0, E10.1; E10.2-E10.8; E10.9 E11.0; E11.1-E11.9

**Moderate/severe renal disease**

ICD-10: I12; I13; N00-N05; N07; N11; N14; N17-N19; Q61

**Gastrointestinal disease**

Ulcer disease K22.1; K25-K28

Mild liver disease: ICD 10: B18; ~~K70.0-K70.3; K70.9~~; K71; K73; K74; K76.0
Moderate/severe liver disease: ICD-10: B15.0; B16.0; B16.2; B19.0; ~~K70.4~~; K72; K76.6; I85

**Cancer,**

Diagnoses from the Danish Cancer Register from 10 years prior admission/index date to 1 year prior:

ICD-10: C00-C79; C97; D37-D44, D48

### Infection

Signs, symptoms and laboratory values were evaluated for the first 24 hours after arrival to the hospital

Diagnostic tests and microbiological samples were evaluated if the samples were drawn or the tests performed within the first 48 hours after arrival with the exception of radiographic sign of ostitis/osteomyelitis, where time frame was 5 days.

**Adapted CDC/NHSN surveillance definition of health care–associated infection and criteria for specific types of infections in the acute care setting** [22]**. Only the changes from the original criteria are listed below.**

The criteria marked with * are changes to the original CDC/NHSN criteria

**2: PULMONARY**

*Other infections of the lower respiratory tract*

Other infections of the lower respiratory tract must meet at least 1 of the following criteria:

1. Patient has organisms seen on smear or cultured from lung tissue or fluid, including pleural fluid.

2. Patient has a lung abscess or empyema seen during a surgical operation or histopathologic examination.

3. Patient has an abscess cavity seen on radiographic examination of lung.

*4. Patient has purulent pleural fluid

*Pneumonia*

1: At least one of the following X-ray or CT scan, new or progressive:

a. Infiltrate

b. Consolidation

c. Cavitation

AND at least one of the following signs:

a. Fever (>38C) with no other cause

b. Leukopenia (<4,000 WBC/mm3) OR leukocytosis (>= 12,000 WBC/mm3)

c. Altered mental status with no other cause, in ≥ 70 y.o.

AND at least two of the following symptoms:

a. New onset of purulent sputum (*yellow, brown or green coloured) OR change in character of sputum OR increased respiratory secretions OR increased suctioning requirements

b. New onset or worsening cough OR dyspnea (a subjective complaint from the patient registered in the electronic patient journal) OR tachypnea (respiratory rate>25)

c. Rales OR broncial breath sounds

d. Worsening gas exchange: O2 desat (*<90% in patients with known COPD OR <94% in patients without COPD) OR increased O2 requirements OR increased ventilation demand

**Lower respiratory tract infection without pneumonia*

*Same criteria as pneumonia but without an X-ray or CT scan indicating a new or progressive infiltrate, consolidation or cavitation.

**3: URINARY TRACT INFECTION**

*Asymptomatic bacteriuria**We did not identify asymptomatic bacteriuria in the manual chart review

**9: OTHER SITES OF INFECTION**

**EYE, EAR, NOSE, THROAT, OR MOUTH INFECTION**

*Conjunctivitis*

*We did not identify patients with conjunctivitis in the manual chart review

**Alternative definitions of infections in patients with a clinical evident infection, but not conforming to the CDC/NHSN definition:**

Infection was defined as identification of a relevant pathogen by microscopy/culture/polymerase chain reaction, positive serology, pneumonia verified by chest X-ray, infection documented with other imaging techniques, positive urine dip test combined with symptoms of urine tract infection, or as typical clinical symptoms such as erysipelas.

### Immunosuppression

**Defined as one or more of the following before the current admission:**1: A moderate to high intake of immunosuppressant medication (ATC: H02AB* or L00-L04, cumulative defined daily dose ≥ 120 in the preceding 120 days with a compliance rate of 80%)
2: Primary immunodeficiency diseases (ICD-10: D61; D70-D71; D80-D82; D84; E70.3; G11.3)
3: Acquired Immune Deficiency Syndrome defining diseases (ICD-10: B20 – B24; F02.41- F02.44)
4: A newly diagnosed malignancy registered in the Danish Cancer Registry, up to a year prior admission ICD-10: C00-C79; C97; D37-D44, D48)

**A history of alcohol-related conditions**

**Defined as at least one of the following before the current admission:**1: A redeemed prescription of Disulfiram (ATC: N07BB01) from 2007 and onwards
2: ≥2 admissions with a discharge diagnosis of an acute alcohol episode (ICD-10: F10.0-F10.1; T519)
3: At least one admission with a discharge diagnosis of a chronic alcohol related diagnosis (ICD-10: F10.2 -F10.9; E24.4; E52.9A; G31.2; G62.1; G72.1; I42.6; K29.2; K70; K85.2; K86.0; L27.8A; O35.4; P04.3; T50.0A; Z71.4; Z72.1; Z50.2)
4: A registration in The National Register of Alcohol Abuse Treatment.

### Systemic Inflammatory Response Syndrome (SIRS)

**Defined as at least two of the following:**1: Fever >38.0°C or hypothermia <36.0°C
2: Tachycardia >90 beats/minute
3: Tachypnea >20 breaths/minute
4: Leucocytosis >12*10^9^/l or leucopenia <4*10^9^/l
The vital signs were measured at arrival to the hospital, and the leucocyte count within the first 24 hours after admission.

### Organ dysfunction

Sepsis was defined as SIRS plus a documented or suspected infection. Severe sepsis was defined as sepsis and at least one organ dysfunction not previously observed/recognized. We defined organ dysfunction as the occurrence of one of the following affections (the latest value retrieved up to 7 days before the date of admission designated ‘earlier’):

**CNS:** Glasgow Coma Score ≤14 recorded on admission or any kind of CNS affection recorded in the electronic patient records on admission if GCS was missing. If pre-existing dementia was known, then deterioration from the preceding usual state was required.)

**Renal:**  (S-creatinine >177 μmol/L and >100 μmol/L S-creatinine increase from earlier S-creatinine) or (S-creatinine >177 μmol/L and earlier S-creatinine <130 μmol/L or never previously registered),

**Gastrointestinal Tract:** S-bilirubin >42 μmol/L and earlier S-bilirubin <43 μmol/L or never previously registered,

**Coagulation** (platelet count <101*10^9^/L and earlier platelet count >100*10^9^/L or never previously registered) or (INR >1.59 and earlier INR <1.60 or never previously registered, without the use of Warfarin),

**Perfusion** lactate >2.5 mmol/L or pH <7.31,

**Respiratory** oxygen saturation <92% recorded at arrival or arterial O_2_ tension <9.75 kPa. Arterial O_2_ saturation <0.92 if oxygen saturation was missing.

**Septic shock** was initially defined as sepsis plus a systolic blood pressure of ≤90 mmHg despite fluid resuscitation of at least 2000mL for at least 1 hour or the use of vasopressor agents.

However, due to inadequate information on initial fluid therapy, we redefined the definition of septic shock to be the occurrence of sepsis plus a systolic blood pressure of ≤90 mmHg and a lactate value >4.0 mmol/L within 4 hours after arrival to the hospital, or the use of vasopressor agents within the first 24 hours after arrival.

### Community-acquired bacteremia

Community-acquired bacteremia was defined, as having a positive blood culture drawn within the first two days of admission. A blood culture consisted of two blood culture sets (each comprising one aerobic and one anaerobic bottle) and we defined bacteraemia as either: (1) recognised pathogens detected in ≥1 blood culture, or (2) common skin contaminants (coagulase-negative staphylococci, *Bacillus* spp, *Propionibacterium* spp, *Corynebacterium* spp, viridans group streptococci, *Aerococcus* spp, or *Micrococcus* spp) detected in ≥2 blood culture sets within 5 days. The date of the first positive blood culture set was regarded as the date of bacteraemia. Polymicrobial bacteraemia was defined as isolation of ≥2 different microorganisms, deemed to represent bacteraemia, within 2 days.

*Microbiological methods*

The blood cultures were incubated and screened for growth of microorganisms for 6 days or until detected positive, using the Bactec 9240 system (Becton Dickinson, NJ, USA) until January 2011 and the Bact/Alert system (BioMérieux) thereafter. Routine methods for identification of bacteria were based on conventional characterisation, the Danish reference programme (http://www.dskm.dk), and automated identification using Vitek 2 (bioMérieux) and MALDI-TOF (SARAMIS, bioMérieux).
